# Interplay of Vitamin D3, Wnt/β-Catenin Pathway, and Oxidative DNA Injury in CMS-Induced Depression Model

**DOI:** 10.3390/biomedicines14050977

**Published:** 2026-04-24

**Authors:** May M. Alrashed, Hajera Tabassum, Dara Aldisi, Maha H. Alhussain, Sadia Arjumand, Mahmoud M. A. Abulmeaty

**Affiliations:** 1 Chair of Medical and Molecular Genetics Research, Department of Clinical Laboratory Sciences, College of Applied Medical Sciences, King Saud University, P.O. Box 10219, Riyadh 11362, Saudi Arabia; 2 Department of Community Health Sciences, College of Applied Medical Sciences, King Saud University, P.O. Box 10219, Riyadh 11362, Saudi Arabia; daldisi@ksu.edu.sa (D.A.); mabulmeaty@ksu.edu.sa (M.M.A.A.); 3 Department of Food Science and Nutrition, College of Food and Agricultural Sciences, King Saud University, Riyadh 11451, Saudi Arabia; mhussein@ksu.edu.sa; 4 Department of Clinical Laboratory Sciences, College of Applied Medical Sciences, King Saud University, P.O. Box 10219, Riyadh 11362, Saudi Arabia; sarjo@ksu.edu.sa

**Keywords:** Wnt/β-catenin signaling, oxidative stress, DNA damage, CMS

## Abstract

**Background/Objectives**: Chronic Mild Stress (CMS) provokes neuroendocrine dysregulation and oxidative injury that compromise neuronal integrity and plasticity. Disruption of the canonical Wnt/β-catenin signaling pathway has been increasingly linked to stress-induced neurobiological dysfunction. Vitamin D3, a neuroactive hormone with antioxidant and immunomodulatory properties, may exert neuroprotection through modulation of this pathway and attenuation of oxidative damage. The study aims to investigate whether vitamin D3 mitigates CMS-induced alterations in Wnt/β-catenin signaling, oxidative stress markers, and oxidative DNA damage in male Wistar rats. **Methods**: Thirty-two male Wistar rats were randomly allocated into four groups (*n* = 8/group): control, CMS only, CMS + vitamin D3 (1000 IU/kg), and CMS + vitamin D3 (10,000 IU/kg). Vitamin D3 was administered intramuscularly three times weekly for 28 days. Hippocampal mRNA expression of Wnt pathway components and brain-derived neurotrophic factor (BDNF) was quantified by RT-qPCR using the 2^−ΔΔCt^ method. Oxidative stress was evaluated by measuring malondialdehyde, glutathione, superoxide dismutase, and catalase, while DNA damage was assessed via 8-OHdG ELISA. **Results**: CMS significantly downregulated Wnt1, β-catenin, and Axin2 mRNA expression (*p* < 0.05) while markedly upregulating GSK-3β (*p* < 0.001). Expression of BDNF was also reduced (*p* < 0.05). Biochemically, CMS increased MDA and 8-OHdG levels (both *p* < 0.001) and decreased glutathione (*p* < 0.001), superoxide dismutase, and catalase activities (*p* < 0.05). Vitamin D3 supplementation significantly reversed these transcriptional and biochemical alterations, restoring β-catenin signaling, improving antioxidant defenses, and reducing oxidative and genotoxic damage. **Conclusions**: Vitamin D3 confers significant neuroprotection under chronic stress by modulating Wnt/β-catenin signaling and attenuating oxidative and DNA damage, thereby enhancing neuronal resilience to prolonged stress exposure.

## 1. Introduction

Stress is a fundamental physiological response that allows organisms to adapt to environmental and internal challenges. However, when stress is chronic, or prolonged, it becomes maladaptive, leading to neurobiological disturbances that compromise neuronal integrity, plasticity, and cognitive performance [1]. The Chronic Mild Stress (CMS) paradigm is widely used in rodents to model prolonged stress exposure, as it recapitulates behavioral, endocrine, and molecular alterations characteristic of stress-induced neuronal dysfunction [2,3]. Sustained stress disrupts key intracellular signaling pathways essential for neuronal health, with the Wnt (Wingless/Integrated)/β-catenin pathway being particularly vulnerable. The hippocampus, central to learning, memory, and emotional regulation, is especially susceptible to these stress-induced molecular disturbances [4,5]. Among the key neuromodulatory networks that safeguard brain function, the canonical Wnt/β-catenin pathway occupies a central position in hippocampal development, maintenance of adult neural circuitry, and lifelong regulation of synaptic plasticity [6]. Perturbation of this pathway destabilizes synaptic architecture, impairs neurogenesis, weakens cellular stress resilience, and renders neurons more vulnerable to oxidative injury [7]. Under physiological conditions, Wnt1-driven signaling promotes neuronal survival and redox balance by suppressing the Axin2/glycogen synthase kinase-3β (GSK-3β) destruction complex, thereby stabilizing β-catenin and enabling transcription of neuroprotective and antioxidant genes, including BDNF and SOD [6,8]. Conversely, chronic stress states disrupt Wnt/β-catenin signaling, accelerate β-catenin degradation, and amplify neuronal susceptibility to structural and oxidative damage [9].

Oxidative stress is a central mediator of stress-induced neuronal dysfunction. Chronic stress elevates reactive oxygen species (ROS), compromises antioxidant defenses, and induces DNA damage, including 8-hydroxy-2′-deoxyguanosine (8-OHdG), a marker of oxidative DNA damage [10,11]. Wnt/β-catenin signaling interacts with oxidative pathways, as β-catenin stabilization promotes transcription of antioxidant genes, while pathway suppression amplifies oxidative injury [12]. Importantly, brain-derived neurotrophic factor (BDNF), a key regulator of neuronal survival, synaptic plasticity, and antidepressant responses, is also a downstream target of canonical Wnt/β-catenin signaling [13]. Stress-induced suppression of Wnt activity contributes to reduced BDNF transcription, thereby weakening neurotrophic support in the hippocampus [14]. Conversely, restoration of Wnt/β-catenin signaling enhances BDNF expression, promoting neurogenesis and synaptic resilience under stress conditions. This mechanistic link positions BDNF as a functional downstream effector through which Wnt signaling modulates hippocampal plasticity during chronic stress.

Vitamin D3 (cholecalciferol) is a neuroactive molecule with established roles in regulating oxidative stress and intracellular signaling, and accumulating evidence indicates that vitamin D3 supplementation can mitigate stress-induced neuroendocrine disturbances [15,16,17]. However, despite these advances, the precise mechanistic interplay between vitamin D3, canonical Wnt1/β-catenin signaling, oxidative stress, and oxidative DNA damage under conditions of chronic stress remains incompletely understood. To address this critical gap, the present study was designed as a mechanistic follow-up to investigate downstream molecular changes in Wnt/β-catenin signaling and oxidative DNA damage within a well-established CMS paradigm with a partially randomized stress schedule. Accordingly, the study assessed mRNA expression of key Wnt1/β-catenin components, evaluated oxidative stress markers and DNA damage, and examined the modulatory effects of vitamin D3 supplementation in rats exposed to CMS, thereby providing mechanistic insight into its role in promoting hippocampal neuroplasticity and neuronal resilience under chronic stress.

## 2. Materials and Methods

### 2.1. Animals, Study Design, and Ethics

Thirty-two adult male Wistar rats (8–10 weeks old; 200–250 g) were used in this study. Animals were housed in the Biomedical Animal Research Unit, College of Applied Medical Sciences, King Saud University, under standard laboratory conditions, including a 12 h light/dark cycle at 25 ± 2 °C, with ad libitum access to chow and water. Animals were housed in standard polypropylene cages (45 × 25 × 15 cm), with four rats per cage. All cage mates belonged to the same experimental group to minimize inter-cage variability and potential social or environmental confounding effects. All animals were handled in accordance with established guidelines for the care and use of laboratory animals and in compliance with the ARRIVE 2.0 guidelines [18]. The experimental protocol was approved by the Animal Research Ethics Committee of King Saud University (Ref. No. KSU-SE-22-24; 24 March 2022). Following a one-week acclimatization period, rats were randomly assigned to four groups (n = 8 per group). Sample size was determined using G*Power software (version 3.1.9.4; effect size = 0.8; α = 0.05; power = 0.95). Male rats were selected to avoid variability associated with estrous cycle-related hormonal fluctuations, which may affect glucose metabolism and oxidative stress outcomes. Animals of comparable age, body weight, and physiological status were included, and all experimental procedures, including housing, diet, and handling, were standardized to reduce variability.

The experimental groups were as follows:

I.     Control: received intramuscular (IM) saline without exposure to stress.
II.   CMS: subjected to the chronic mild stress protocol for 28 days without treatment.
III. CMS + vitamin D3 (1000 IU/kg): received vitamin D3 via IM injection daily for 28 days during CMS exposure.
IV.   CMS + vitamin D3 (10,000 IU/kg): received vitamin D3 via IM injection daily for 28 days during CMS exposure.

Adherence to the ARRIVE checklist ensured transparent reporting of the experimental design, justification of sample size, randomization and blinding procedures, and measures taken to safeguard animal welfare.

### 2.2. Inducing Chronic Mild Stress

Rats in Groups II–IV underwent a 28-day Chronic Mild Stress (CMS) regimen, with partially randomized stress schedule, modified from previously published protocols [15]. To induce depression-like behaviors, animals were exposed to a variety of mild stressors applied in a random sequence, including cage tilting, cage shaking, wet bedding, continuous light exposure, and temporary food or water deprivation. The complete schedule of stressors is presented in Table 1. Successful induction of the CMS model and its behavioral characterization were previously established and validated in our earlier work [15], which included standard behavioral assessments such as the Sucrose Preference Test, Forced Swim Test, Tail Suspension Test, Open Field Test, and Elevated Plus Maze. Behavioral assessments were not repeated in the present study, as the induction of the CMS model and its associated behavioral effects had already been comprehensively validated in that study. Accordingly, the present study was designed as a mechanistic follow-up to investigate downstream molecular changes in Wnt/β-catenin signaling and oxidative DNA damage under the established stress paradigm.

### 2.3. Administration of Vitamin D3

Vitamin D3 (Memphis Company for Pharmaceutical and Chemical Industries, Cairo, Egypt; 100,000 IU/mL, 2 mL ampule) was diluted in 0.9% saline to achieve the required doses. IM injections were administered three times per week during the 28-day CMS period [19]. Group III received 1000 IU/kg, and Group IV received 10,000 IU/kg. The control group received equivalent volumes of saline injections. IM administration was selected to ensure reliable systemic delivery and consistent dosing compared with other routes. To minimize local tissue irritation and improve animal welfare, injections were performed in the hind limb muscles using a limited volume (0.2–0.6 mL per rat per injection), and injection sites were alternated throughout the study period. The injection schedule was distributed across the week (Sunday and Tuesday injections covering two days, and Thursday injections covering three days) to maintain continuous supplementation over weekends. The selected doses were based on previously published studies [20,21].

### 2.4. Body Weight Monitoring and Sample Collection

Body weight was recorded at baseline and monitored regularly throughout the experiment to assess general health and physiological stress. At the end of the study, rats were euthanized under isoflurane anesthesia, and blood samples were collected via cardiac puncture. Serum was separated by centrifugation and stored at −80 °C, while hippocampal tissues were rapidly dissected and preserved for molecular analyses.

### 2.5. Serum Vitamin D3 and Corticosterone Levels

Serum vitamin D3, and corticosterone were measured using ELISA, following manufacturers’ protocols, as previously reported for this cohort of CMS-exposed rats [22]. Vitamin D3 levels were assessed using the Rat 25-Dihydroxy Vitamin D ELISA Kit (MyBioSource, San Diego, CA, USA; Catalog: MBS2601819), and serum corticosterone was quantified using the Rat Corticosterone ELISA Kit (MyBioSource, San Diego, CA, USA; catalog: MBS761865.

### 2.6. Hippocampal Gene Expression Analysis by Quantitative Real-Time PCR (qRT-PCR)

Frozen hippocampal tissues stored at −80 °C were utilized for gene expression studies. Prior to RNA extraction, tissues were carefully handled on ice to preserve RNA integrity.

#### 2.6.1. RNA Isolation and Quality Assessment

Total RNA was isolated from hippocampal samples using the PureLink™ RNA Mini Kit (Invitrogen, Thermo Fisher Scientific, Pleasanton, CA, USA) following the manufacturer’s protocol. Tissues were homogenized in lysis buffer on ice, and RNA was bound to a silica-based spin column. After sequential washes to remove contaminants, RNA was eluted in RNase-free water. The concentration and purity of RNA were determined spectrophotometrically using a NanoDrop 2000 (Thermo Scientific, USA), and only samples with an A260/A280 ratio between 1.8 and 2.0 were advanced for cDNA synthesis. Isolated RNA was stored at −80 °C until further processing.

#### 2.6.2. cDNA Synthesis

Complementary DNA (cDNA) was synthesized using the High-Capacity cDNA Reverse Transcription Kit (Applied Biosystems, Pleasanton, CA, USA). For each reaction, 1 µg of total RNA was reverse-transcribed in a 20 µL reaction volume according to the manufacturer’s instructions, under the following cycling conditions: 25 °C for 10 min, 37 °C for 120 min, and 85 °C for 5 min, using a Veriti Thermal Cycler (Applied Biosystems, Thermo Fisher Scientific, USA). The resulting cDNA was stored at −20 °C until qPCR analysis.

#### 2.6.3. Quantitative Real-Time PCR Amplification

qPCR was performed using Power SYBR™ Green PCR Master Mix (Applied Biosystems, Thermo Scientific, USA) on a ViiA 7 Real-Time PCR System (Applied Biosystems, Thermo Scientific, USA). Each 20 µL reaction contained 1 µL cDNA, 10 µL SYBR Green Master Mix, 1 µL each of forward and reverse primers (Macrogen, Seoul, Republic of Korea), and 7 µL nuclease-free water. Thermal cycling parameters included an initial denaturation at 95 °C for 10 s, followed by 40 cycles of 95 °C for 10 s and 60 °C for 30 s. A melting curve analysis was conducted at the end of each run to confirm amplification specificity.

#### 2.6.4. Genes of Interest and Data Quantification

Genes analyzed in this study included Wnt1, β-catenin, GSK-3β, and Axin2 to evaluate canonical Wnt signaling, along with BDNF to assess neurotrophic support and synaptic plasticity. Primer sequences are provided in Table 2. GAPDH was used as the internal reference for normalization. Relative gene expression levels were calculated using the 2^−ΔΔCt^ method.

### 2.7. Assessment of Oxidative Damage

#### 2.7.1. Lipid Peroxidation (Malondialdehyde; MDA) Assay

Lipid peroxidation was quantified by determining malondialdehyde (MDA) levels using a commercial MDA Assay Kit (ab118970; Abcam, Cambridge, UK) according to the manufacturer’s instructions. In brief, brain tissue (10 mg) was homogenized in 303 µL of ice-cold MDA lysis solution consisting of 300 µL MDA Lysis Buffer and 3 µL butylated hydroxytoluene (BHT) to prevent artificial oxidation during processing. The homogenate was centrifuged at 13,000× *g* for 10 min at 4 °C, and the resulting supernatant was collected for analysis. For the assay, 200 µL of each sample or MDA standard was mixed with 600 µL of thiobarbituric acid (TBA) solution and incubated at 95 °C for 60 min to form MDA–TBA adducts. The reaction was terminated by rapid cooling on ice, followed by centrifugation to remove any precipitate. The absorbance of the supernatant was measured at 532 nm using a microplate reader (Sunrise™, Tecan Group Ltd., Mannedorf, Switzerland). A standard curve was generated using MDA concentrations ranging from 20–100 µM. MDA levels were expressed as nmol/mL of brain homogenate.

#### 2.7.2. Analysis of Antioxidant Markers

##### Glutathione Assay

Reduced glutathione (GSH) levels were quantified using the Glutathione Assay Kit (CS0260-1KT, Sigma-Aldrich, St. Louis, MO, USA), following the manufacturer’s protocol. Brain tissue (10 g) was homogenized in 10 volumes of ice-cold 5% 5-sulfosalicylic acid (SSA) until a uniform suspension was achieved. The homogenates were maintained at 2–8 °C for 10 min and centrifuged at 10,000× *g* for 10 min at 4 °C. The resulting supernatant was collected and used immediately for analysis. The assay is based on the reduction of 5,5′-dithiobis (2-nitrobenzoic acid) (DTNB) by GSH to form 5-thio-2-nitrobenzoic acid (TNB), measured spectrophotometrically at 412 nm using a Tecan Sunrise microplate reader (Tecan, Austria). GSH concentrations were calculated from a standard curve and expressed as µg/mL of brain homogenate.

##### Antioxidant Enzymes: Superoxide Dismutase (SOD) and Catalase (CAT)

Superoxide dismutase activity was quantified using the SOD Assay Kit-WST (19160, Sigma-Aldrich, USA) according to the manufacturer’s protocol. Approximately 10 mg of brain tissue was homogenized in 200 µL of the assay buffer supplied with the kit and centrifuged at 10,000× *g* for 15 min at 4 °C. The clear supernatant was collected for analysis. The assay is based on the reduction in the water-soluble tetrazolium salt (WST-1) by superoxide anions, which generates a colored formazan dye measurable at 450 nm. SOD activity was calculated by determining the degree of inhibition of this colorimetric reaction. Absorbance was measured using a Tecan Sunrise microplate reader, and enzyme activity was expressed as inhibition rate %.

Catalase activity was determined using the Catalase Activity Assay Kit (ab83464, Abcam, UK) in accordance with the manufacturer’s instructions. The same supernatant prepared for SOD analysis was used. Samples were incubated with hydrogen peroxide (H_2_O_2_) at 25 °C, followed by addition of stop solution and developer. Catalase decomposed H_2_O_2_, and the remaining H_2_O_2_ reacts with the colorimetric probe to generate a measurable signal. Absorbance was recorded at 570 nm, and catalase activity was expressed as mIU/mL. The assay had a limit of detection of 1 µU and a detection range of 1–100 µU.

### 2.8. Assessment of DNA Damage by Measuring 8-Hydroxy-2′-Deoxyguanosine (8-OHdG)

Brain tissue (10 mg) was homogenized in ice-cold phosphate-buffered saline (PBS, pH 7.4) containing 0.1% Triton X-100, followed by centrifugation at 10,000× *g* for 10 min at 4 °C. The resulting supernatant was used to determine free 8-OHdG levels using a competitive ELISA as previously described [23]. The level of oxidative DNA damage was quantified using the 8-OHdG ELISA Kit (ab201734, Abcam, UK) according to the manufacturer’s protocol. Absorbance was recorded at 450 nm using a microplate reader (IRE96, SFRI, Berganton, France), and results were expressed as ng/mL of brain homogenate.

### 2.9. Statistical Analysis

All data were analyzed using Sigmaplot (software version 12). Group comparisons were performed using one-way ANOVA followed by the Holm–Sidak post hoc test, which includes correction for multiple comparisons. A *p*-value < 0.05 was considered statistically significant.

## 3. Results

### 3.1. Body Weight and Hormonal Profiles in Response to CMS and Vitamin D3 Administration

Table 3 summarizes the effects of CMS and vitamin D3 treatment on body weight. Baseline body weights were comparable across all groups, with no significant differences observed. After 4 weeks, the CMS group showed a significant reduction in final body weight compared to the control group (* *p* < 0.05), indicating stress-induced metabolic alteration. No comparisons with baseline values were performed, as no significant differences were observed at baseline. Statistical analysis was performed on final body weights between groups using one-way ANOVA followed by the Holm–Sidak post hoc test. Treatment with vitamin D3 at 1000 IU/kg resulted in a significant increase in final body weight compared to the CMS group (* *p* < 0.05), indicating partial recovery toward control levels. In contrast, the high-dose vitamin D3 group (10,000 IU/kg) showed no significant difference in final body weight compared to the CMS group.

Serum vitamin D3 concentrations declined from 35.33 ± 2.50 ng/mL in controls to 32.52 ± 3.10 ng/mL (*p* < 0.001) in the CMS group, while vitamin D3 (10,000 IU/kg) increased levels to 43.86 ± 1.93 ng/mL. Similarly, corticosterone increased significantly under CMS (426.31 ± 13.06 vs. 283.86 ± 23.88 ng/mL in controls; *p* < 0.001), whereas vitamin D3 (10,000 IU/kg) restored values near control levels (276.30 ± 17.55 ng/mL). These measurements were obtained previously in the same cohort and are provided in the Supplementary Files (Table S1).

### 3.2. Vitamin D3 Restores CMS-Induced Dysregulation of Canonical Wnt Signaling Genes

**Figure 1:**
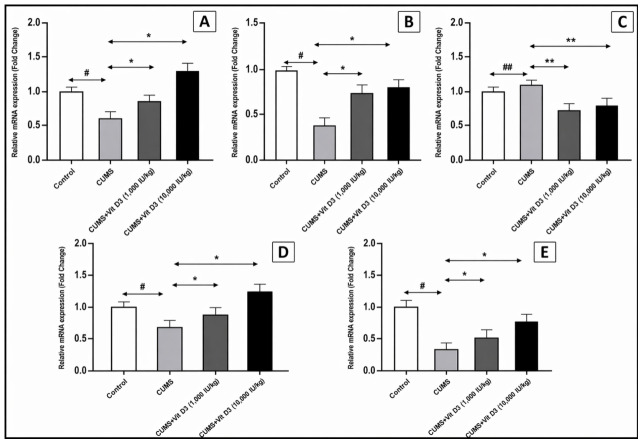
Vitamin D3 modulates hippocampal Wnt/β-catenin signaling components and BDNF disruption in CMS. Relative mRNA expression levels of (**A**) Wnt1, (**B**) β-catenin, (**C**) GSK-3β, (**D**) Axin-2, and (**E**) BDNF in the hippocampus of the control, CMS, and vitamin D3-treated groups. Data are presented as mean ± SEM and expressed as fold change relative to the control group (set at 1). Statistical analysis was performed using one-way ANOVA followed by Holm–Sidak post hoc tests. # indicates *p* < 0.05 and ## *p* < 0.001 for CMS versus control; * indicates *p* < 0.05 and ** *p* < 0.001 for the vitamin D3-treated groups versus CMS. CMS induced marked downregulation of Wnt signaling pathway genes and BDNF, except GSK-3β, which was significantly upregulated. Vitamin D3 administration reversed these effects in a dose-dependent manner, with the 10,000 IU/kg dose producing the most pronounced improvement.

CMS exposure induced a substantial disruption of the Wnt/β-catenin signaling pathway, reflected by significant down-regulation of its core transcriptional components (Figure 1A–D). Relative to the control group, CMS decreased Wnt1 mRNA expression to 0.45 ± 0.22-fold (*p* < 0.05), suppressed β-catenin to 0.68 ± 0.20-fold (*p* < 0.05), and markedly reduced the downstream effector Axin2 to 0.39 ± 0.12-fold (*p* < 0.05). In contrast, the inhibitory kinase GSK-3β was significantly up-regulated to 1.16 ± 0.28-fold compared with controls (*p* < 0.001). Vitamin D3 treatment demonstrated a clear dose-dependent restorative effect. The 1000 IU/kg dose significantly increased Wnt1, β-catenin, and Axin2 expression (*p* < 0.05 for all) and decreased GSK-3β (*p* < 0.001) relative to CMS. The higher 10,000 IU/kg dose produced an even stronger correction, further enhancing Wnt pathway gene expression and suppressing GSK-3β (*p* < 0.001). Overall, significant differences were observed between the control and CMS groups and between the CMS and both vitamin D3-treated groups (Wnt1, β-catenin, Axin2: *p* < 0.05; GSK-3β: *p* < 0.001), indicating that vitamin D3 effectively restores transcriptional balance and counteracts stress-induced pathway suppression in a dose-dependent manner.

### 3.3. Vitamin D3 Rescues Wnt1–BDNF Neurotrophic Signaling Impaired by CMS

BDNF mRNA expression, previously reported in this same cohort [18], was further examined here in the context of Wnt pathway regulation (Figure 1E). CMS produced a marked suppression of hippocampal BDNF, reducing its expression to 0.30 ± 0.04-fold (control set at 1-fold; *p* < 0.05). Vitamin D3 supplementation mitigated this deficit in a dose-dependent manner. The 1000 IU/kg dose partially restored BDNF expression to 0.50 ± 0.04-fold (*p* < 0.05 vs. CMS), while the 10,000 IU/kg dose produced a more substantial recovery, elevating expression to 0.80 ± 0.04-fold (*p* < 0.05 vs. CMS).

### 3.4. Vitamin D3 Reverses CMS-Induced Oxidative Stress Imbalance

**Figure 2:**
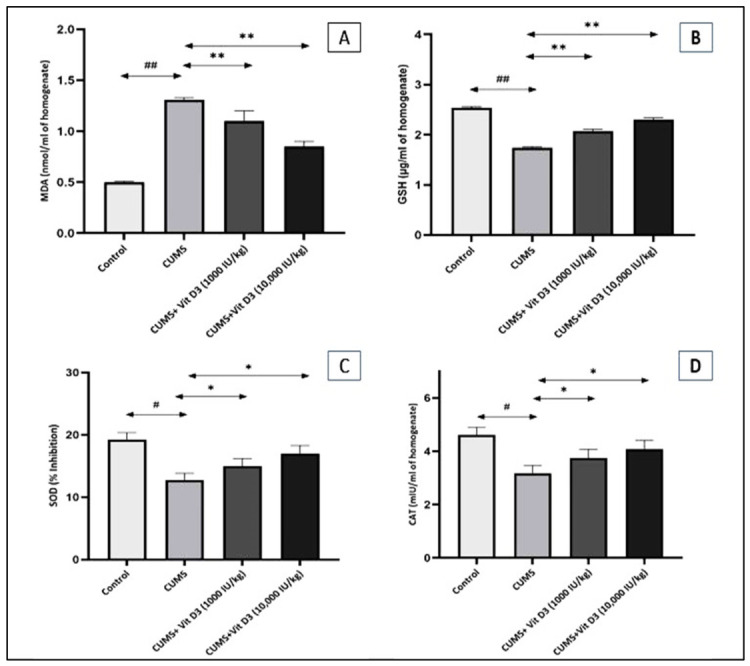
Vitamin D3 mitigates CMS-induced oxidative imbalance by enhancing antioxidant capacity and suppressing lipid peroxidation in the rat brain. Levels of (**A**) MDA, (**B**) GSH, (**C**) SOD, and (**D**) CAT in brain homogenates of the control, CMS, and vitamin D3-treated groups. Data are presented as mean ± SEM. Statistical analysis was performed using one-way ANOVA followed by Holm–Sidak post hoc tests. # indicates *p* < 0.05 and ## *p* < 0.001 for CMS versus control; * indicates *p* < 0.05 and ** *p* < 0.001 for the vitamin D3-treated groups versus CMS. CMS increased lipid peroxidation (MDA) while reducing antioxidant defenses (GSH, SOD, and CAT). Vitamin D3 produced a dose-dependent reversal, lowering MDA and restoring GSH, SOD, and CAT activities.

Chronic exposure to CMS produced significant alterations in oxidative stress markers compared with the control group (Figure 2A–D). MDA levels were markedly elevated in the CMS group (1.31 ± 0.02 nmol/mL) relative to controls (0.50 ± 0.01 nmol/mL; *p* < 0.001), indicating substantial lipid peroxidation. Treatment with vitamin D3 reduced this elevation in a dose-dependent manner, with the 1000 IU/kg dose lowering MDA to 1.10 ± 0.10 nmol/mL (*p* < 0.001 vs. CMS) and the 10,000 IU/kg dose further reducing levels to 0.85 ± 0.05 nmol/mL (*p* < 0.001 vs. CMS).

Similarly, GSH concentrations were significantly decreased in CMS-exposed rats (1.74 ± 0.02 µg/mL) compared with the control group (2.54 ± 0.02 µg/mL; *p* < 0.001), reflecting impaired antioxidant defense. Vitamin D3 supplementation significantly restored GSH levels, with values rising to 2.07 ± 0.04 µg/mL in the 1000 IU/kg group (*p* < 0.001 vs. CMS) and 2.30 ± 0.04 µg/mL in the 10,000 IU/kg group (*p* < 0.001 vs. CMS). Furthermore, CMS significantly reduced SOD activity (12.75 ± 1.10% inhibition) in comparison with control animals (19.25 ± 1.10% inhibition; *p* < 0.05). Vitamin D3 treatment led to a partial recovery of SOD activity, reaching 15.00 ± 1.20% inhibition in the 1000 IU/kg group and 17.00 ± 1.29% in the 10,000 IU/kg group (*p* < 0.05 vs. CMS for both). Additionally, CAT activity was significantly suppressed in the CMS group (3.17 ± 0.30 mIU/mL) relative to controls (4.62 ± 0.28 mIU/mL; *p* < 0.05). Vitamin D3 administration improved CAT levels to 3.75 ± 0.32 mIU/mL at 1000 IU/kg and 4.07 ± 0.33 mIU/mL at 10,000 IU/kg (*p* < 0.05 vs. CMS). Overall, these results demonstrate that vitamin D3 effectively counteracts CMS-induced oxidative imbalance across multiple antioxidant systems in a dose-dependent manner.

### 3.5. Vitamin D3 Alleviates CMS-Induced Oxidative DNA Injury

**Figure 3:**
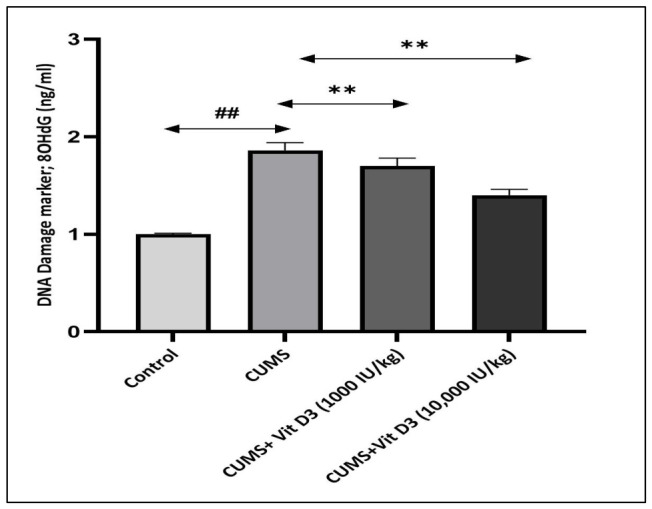
Vitamin D3 attenuates CMS-induced oxidative DNA damage in the rat brain. Levels of 8-OHdG in brain homogenates of the control, CMS, and vitamin D3-treated groups. Data are presented as mean ± SEM. Statistical analysis was performed using one-way ANOVA followed by Holm–Sidak post hoc tests. ## *p* < 0.001 for CMS versus control and ** *p* < 0.001 for vitamin D3-treated groups versus CMS. CMS elicited a significant rise in oxidative DNA damage, whereas vitamin D3 produced a significant reduction in 8-OHdG compared with CMS.

Chronic exposure to CMS significantly elevated hippocampal 8-OHdG levels, increasing from 1.56 ± 0.01 ng/mL in controls to 1.86 ± 0.08 ng/mL (*p* < 0.001) (Figure 3). Vitamin D3 supplementation reduced this elevation in a dose-dependent manner. Vitamin D3 (1000 IU/kg) lowered 8-OHdG to 1.70 ± 0.08 ng/mL, while vitamin D3 (10,000 IU/kg) further decreased it to 1.40 ± 0.06 ng/mL (*p* < 0.001). These results indicate that vitamin D3 provides measurable protection against CMS-induced oxidative DNA damage.

## 4. Discussion

The present study demonstrates that CMS paradigm with partially randomized stressors induces robust molecular, biochemical, and endocrine alterations in the hippocampus, leading to significant disruption of canonical Wnt/β-catenin signaling, neurotrophic support, and redox homeostasis. Exposure to CMS significantly downregulated hippocampal transcription of Wnt1, β-catenin, and Axin2 (all *p* < 0.05), while markedly upregulating the inhibitory kinase GSK-3β (*p* < 0.001). These transcriptional disturbances were accompanied by a pronounced reduction in BDNF expression (*p* < 0.05), a significant increase in lipid peroxidation (*p* < 0.001), and marked impairments in antioxidant defenses, including reduced GSH, SOD, and CAT activities (*p* < 0.05). In parallel, CMS significantly elevated hippocampal levels of the oxidative DNA damage marker, 8-OHdG (*p* < 0.001), indicating increased genomic oxidative injury. Importantly, vitamin D3 supplementation significantly reversed these alterations by restoring Wnt/β-catenin pathway activity, enhancing BDNF expression, reducing oxidative stress, and attenuating DNA damage (*p* < 0.05 to *p* < 0.001), supporting a coordinated neuroprotective role for vitamin D3 in preserving hippocampal transcriptional stability and neuronal resilience under chronic stress.

The physiological and endocrine changes observed in the CMS group further support sustained activation of the hypothalamic–pituitary–adrenal (HPA) axis as a core component of the stress response [24]. The reduction in body-weight gain and the marked elevation in circulating corticosterone levels are consistent with chronic glucocorticoid exposure, which disrupts energy balance by suppressing appetite and altering metabolic regulation. Vitamin D3 at 1000 IU/kg partially reversed CMS-induced weight loss, suggesting a protective effect against stress-related metabolic dysregulation, likely via modulation of endocrine function and partial normalization of HPA axis activity. In contrast, 10,000 IU/kg showed no significant benefit, indicating a possible dose-dependent or threshold effect. Overall, moderate vitamin D3 supplementation appears to confer partial protection by restoring systemic metabolic and endocrine homeostasis (Table 3). Furthermore, CMS significantly reduced serum vitamin D3 concentrations (*p* < 0.01), indicating a pronounced endocrine imbalance that may further exacerbate neural vulnerability. Vitamin D3 supplementation, particularly at 10,000 IU/kg, significantly normalized corticosterone levels (*p* < 0.001) (Table S1). The associated increase in circulating vitamin D3 confirms adequate systemic bioavailability and suggests that normalization of HPA axis activity may contribute to the downstream molecular and biochemical effects observed in the present study.

At the transcriptional level, CMS induced a pronounced impairment of canonical Wnt/β-catenin signaling (Figure 1A–D). Hippocampal expression of Wnt1, β-catenin, and Axin2 was reduced to approximately 45%, 68%, and 39% of control levels, respectively (*p* < 0.05), while GSK-3β expression increased to 116% of control (*p* < 0.001). This expression profile is indicative of sustained activation of the β-catenin destruction complex and impaired Wnt-dependent transcription. Aberrant activation of GSK-3β and destabilization of β-catenin are increasingly recognized as central molecular drivers of stress-induced neural vulnerability. Consistent with this interpretation, Fatima et al., 2019, demonstrated CMS-associated upregulation of GSK-3β and suppression of β-catenin in stress-sensitive brain regions, including the hippocampus and prefrontal cortex [25]. Similarly, El-Kadi et al., 2024, reported that chronic stress diminishes hippocampal β-catenin availability, enhances destruction complex activity, and suppresses BDNF expression, molecular changes directly linked to impaired neurogenesis and behavioral deficits [26].

Vitamin D3 supplementation robustly counteracted these stress-induced transcriptional abnormalities in a dose-dependent manner. Administration of vitamin D3 at 1000 IU/kg significantly increased Wnt1, β-catenin, and Axin2 expression to 148.9%, 130.9%, and 192.3% of CMS values, respectively (*p* < 0.05), while reducing GSK-3β expression to 64.7% of CMS levels (*p* < 0.001). A more pronounced normalization was observed with the higher dose (10,000 IU/kg), which further elevated Wnt1, β-catenin, and Axin2 expression to 166.7%, 202.9%, and 205.1% of CMS values, respectively (*p* < 0.05), while maintaining significant suppression of GSK-3β to 75% of CMS levels (*p* < 0.001). These findings support functional cross-talk between vitamin D3 signaling and the Wnt/β-catenin pathway and identify vitamin D3 as a potent modulator of stress-sensitive transcriptional programs. The ability of vitamin D3 to restore Wnt pathway integrity aligns with accumulating evidence that pharmacological reactivation of canonical Wnt/β-catenin signaling can reverse stress-induced molecular and structural deficits. Xiao et al., 2021, demonstrated that CMS suppresses multiple Wnt pathway components and impairs hippocampal neurogenesis, whereas Baicalin treatment reinstated β-catenin signaling, promoted its nuclear translocation, and upregulated downstream neurogenic targets [27]. Experimental studies using Vilazodone, Rebamipide, and 3-O-methylquercetin have similarly shown that stabilization of β-catenin or inhibition of GSK-3β enhances hippocampal neurogenesis, improves cognitive performance, and mitigates neurodegenerative pathology [26,28,29]. Notably, the regulatory effects of vitamin D3 on Wnt signaling appear to be context dependent. Zou et al., 2024, reported that vitamin D3 suppresses pathological overactivation of the Wnt/β-catenin pathway in autoimmune disease models [30]. When considered alongside the present findings, this bidirectional modulation suggests that vitamin D3 functions as a homeostatic regulator capable of normalizing Wnt pathway activity according to the underlying pathological state.

**Figure 4:**
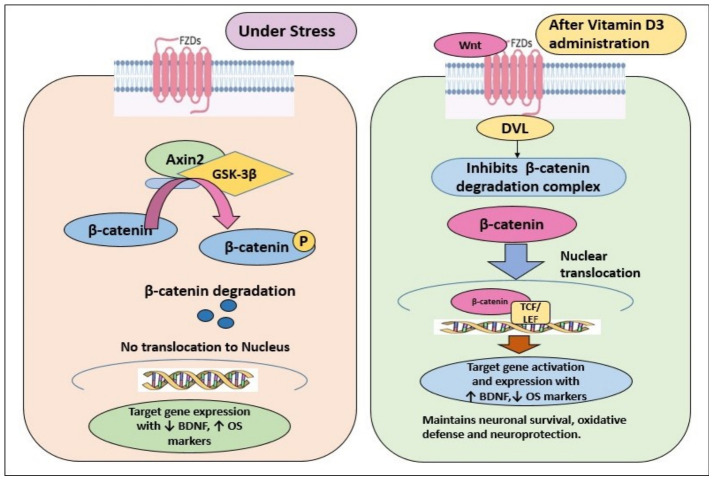
Schematic representation of Canonical Wnt/β-Catenin pathway disruption by CMS and restoration by vitamin D3. The schematic illustrates how chronic stress disrupts Wnt/β-catenin signaling and how vitamin D3 effectively restores its function. Under normal conditions, Wnt1 binds to its receptors and suppresses the GSK-3β/Axin2 destruction complex, allowing β-catenin to accumulate, enter the nucleus, and activate genes that maintain BDNF expression and antioxidant protection. In contrast, CMS imposes a profound inhibitory shift: Wnt1 and Axin2 are downregulated, GSK-3β activity increases, β-catenin is rapidly degraded, and downstream neuroprotective targets, including BDNF, are markedly suppressed, alongside heightened oxidative stress. Vitamin D3 treatment reverses this pattern by enhancing Wnt1, β-catenin, and Axin2 levels, suppressing GSK-3β, restoring or ↑ increasing BDNF, and ↓ reducing oxidative stress. Together, these changes highlight vitamin D3’s capacity to reinstate Wnt pathway integrity and reinforce neuroprotective resilience under chronic stress.

To further elucidate the mechanistic link between chronic stress, Wnt signaling, and neuroprotection, Figure 4 illustrates the canonical Wnt/β-catenin pathway and its modulation by vitamin D3. Activation occurs when Wnt1 binds Frizzled receptors and LRP5/6 co-receptors, recruiting Dishevelled (DVL) and inhibiting the β-catenin destruction complex (GSK-3β and Axin2) [31]. Stabilized β-catenin translocates to the nucleus, activating transcription of genes governing neuronal survival, synaptic plasticity, antioxidant defense, and BDNF expression [32]. Under CMS conditions, this cascade is disrupted through downregulation of Wnt pathway components and sustained activation of the destruction complex, resulting in enhanced β-catenin degradation, suppression of protective gene expression, and increased neuronal vulnerability [26,27]. Vitamin D3 counteracts these disruptions at multiple levels by enhancing Wnt1, β-catenin, and Axin2 expression, suppressing GSK-3β, and restoring BDNF transcription, thereby re-establishing the functional integrity of the Wnt/β-catenin–BDNF axis.

Consistent with this mechanistic framework, CMS exposure resulted in a marked suppression of hippocampal BDNF expression to approximately 30% of control levels (*p* < 0.05) (Figure 1E). Vitamin D3 supplementation partially and dose-dependently reversed this deficit, restoring BDNF expression to approximately 50% and 80% of control values at 1000 and 10,000 IU/kg, respectively (*p* < 0.05). BDNF downregulation is a well-established molecular hallmark of stress-induced hippocampal dysfunction and is closely linked to impaired neurogenesis and synaptic plasticity [5,33,34]. Reciprocal regulation between BDNF–TrkB signaling and the Wnt/β-catenin pathway forms a critical feedback loop that preserves neuronal integrity [35]. Disruption of this axis under CMS conditions likely contributes to hippocampal maladaptation, whereas vitamin D3-mediated restoration of Wnt signaling may secondarily drive BDNF re-expression and support synaptic resilience [36]. Supportingly, Lei et al., 2023, demonstrated that pharmacological inhibition of GSK-3β restores neurotrophic signaling, including BDNF upregulation, and mitigates neurodegenerative pathology, highlighting GSK-3β as a key molecular convergence point linking Wnt signaling to neurotrophic support [36]. In the present study, CMS-induced downregulation of core Wnt pathway genes likely disrupted this regulatory axis, contributing to impaired neurogenesis and hippocampal maladaptation. Vitamin D3 supplementation effectively reinstated Wnt/β-catenin activity, which may secondarily drive BDNF re-expression and reinforce antioxidant defenses, highlighting an integrated signaling network connecting Wnt activation, neurotrophic support, and redox homeostasis. Accordingly, the marked reduction in BDNF under CMS likely reflects the combined consequences of Wnt pathway suppression and oxidative stress. By re-establishing the Wnt/β-catenin–BDNF axis, vitamin D3 appears to counteract stress-induced deficits in synaptic plasticity and neuronal resilience, offering mechanistic insight into its neuroprotective efficacy under chronic stress conditions.

In parallel with transcriptional and neurotrophic disturbances, CMS induced a pronounced disruption of redox homeostasis, evidenced by a 162% increase in MDA levels relative to controls (*p* < 0.001), a 31% reduction in GSH, and impairments in SOD and CAT activities (*p* < 0.05–0.001), reflecting extensive oxidative stress and compromised antioxidant defenses. These observations indicate a severely disturbed redox balance under chronic stress, in line with previous reports by Abulmeaty et al., 2023, and Zou et al., 2020, which documented CMS-associated oxidative damage, mitochondrial dysfunction, and impaired antioxidant capacity [37,38]. Vitamin D3 supplementation exerted a clear and dose-dependent antioxidant effect: MDA levels were reduced by 16% and 35% at 1000 and 10,000 IU/kg, respectively (*p* < 0.001). Non-enzymatic antioxidant reserves improved with GSH increases of 19% and 32%, while enzymatic defenses rose by 18% and 33% for SOD and 18% and 28% for CAT following low- and high-dose vitamin D3, respectively. These findings align with reports demonstrating that attenuation of oxidative stress is a critical determinant of neuroprotection in CMS models [39,40].

Beyond its direct biochemical impact, oxidative stress exerts deleterious effects on intracellular signaling by disrupting transcriptional regulation essential for neuronal adaptation. Notably, oxidative stress potently inhibits canonical Wnt/β-catenin signaling, largely via GSK-3β activation, destabilizing cytosolic β-catenin and repressing transcription of neuroprotective Wnt targets such as Axin [41]. Supporting this mechanism, Yde Ohki et al., 2020, demonstrated that stress-associated oxidative conditions drive GSK-3β-dependent β-catenin destabilization, leading to suppression of Wnt-regulated transcriptional programs critical for neuronal survival [42]. Similarly, Xiao et al., 2021, showed that CMS suppresses hippocampal Wnt/β-catenin signaling, reduces nuclear β-catenin translocation, and downregulates BDNF, whereas restoration of Wnt signaling reversed stress-induced neurogenic and behavioral deficits [27]. Vitamin D3 supplementation effectively interrupted this pathological cascade. By restoring redox homeostasis and reinforcing antioxidant defenses, vitamin D3 alleviated oxidative pressure on redox-sensitive pathways, thereby permitting stabilization of β-catenin and reactivation of downstream transcriptional programs, including BDNF expression. This mechanistic interpretation is further supported by Lisakovska et al., 2023, who demonstrated that glucocorticoid-induced neurotoxicity disrupts the brain’s auto/paracrine vitamin D3 system, leading to oxidative stress and synaptic dysfunction that were reversed by vitamin D3 supplementation [16]. Collectively, these findings position vitamin D3 as a homeostatic regulator capable of counteracting oxidative stress-driven suppression of redox-sensitive signaling networks. By simultaneously reinforcing antioxidant defenses and stabilizing the interconnected Wnt/β-catenin–BDNF axis, vitamin D3 preserves hippocampal integrity and neuronal resilience under chronic stress, underscoring its therapeutic potential in stress-related neuropsychiatric disorders.

Among the molecular perturbations induced by CMS, elevation of 8-OHdG represents a particularly consequential hallmark, directly implicating oxidative DNA damage as a central pathological outcome of sustained stress exposure. In the present study, CMS increased hippocampal 8-OHdG levels by ~30% relative to controls (Figure 3), reflecting accumulation of oxidative lesions within neuronal genomes. Oxidative DNA damage intersects with redox-sensitive signaling pathways that govern neuronal survival and plasticity. Canonical Wnt/β-catenin signaling supports antioxidant defense, DNA repair capacity, and synaptic maintenance; thus, its suppression under CMS establishes a self-perpetuating cycle wherein genomic instability further weakens neuroprotective signaling, accelerating hippocampal vulnerability [25]. Vitamin D3 supplementation effectively interrupted this deleterious cascade. Alongside restoration of redox balance and Wnt pathway gene expression, vitamin D3 significantly attenuated oxidative DNA injury, reducing 8-OHdG by ~10% and ~18% at 1000 and 10,000 IU/kg, respectively (all *p* < 0.001). These findings suggest that vitamin D3 limits stress-induced genotoxicity by restraining GSK-3β activity, stabilizing β-catenin, and re-engaging transcriptional programs that preserve genomic and synaptic integrity, a conclusion strongly supported by the meta-analysis of Goh et al., 2021, which identified 8-OHdG as a consistently elevated biomarker of oxidative DNA damage across multiple psychiatric disorders [11,43]. Collectively, these results identify oxidative DNA damage and suppression of Wnt/β-catenin signaling as interlinked drivers of CMS-induced hippocampal dysfunction.

## 5. Conclusions

The present findings support an integrated mechanistic model in which vitamin D3 attenuates the neurobiological consequences of chronic mild stress through coordinated multisystem regulation. Vitamin D3 restores canonical Wnt/β-catenin signaling and downstream neurotrophic support via BDNF, while concurrently strengthening antioxidant defenses (GSH, SOD, CAT) and reducing oxidative and DNA damage (MDA, 8-OHdG). Together, these convergent actions establish a cellular environment that preserves genomic stability, synaptic plasticity, and neuronal resilience. Collectively, these results position vitamin D3 as a potent modulator of stress-induced neurobiological dysfunction and highlight its promise as an adjunctive therapeutic strategy for stress-related and depressive disorders.

## 6. Limitations and Future Directions

A key limitation of the present study is its reliance solely on mRNA expression data, without accompanying protein-level validation for Wnt/β-catenin pathway components or BDNF. Although transcriptional profiling offers meaningful mechanistic insight, mRNA abundance does not necessarily reflect protein levels, post-translational modifications, or functional signaling activity. This constraint was partly due to limited hippocampal tissue availability and resource restrictions. Future work should incorporate measurements of total and active (non-phosphorylated) β-catenin, GSK-3β phosphorylation status, nuclear β-catenin localization, downstream transcriptional activity, and oxidative-stress-related proteins, along with sex-based comparisons to capture potential biological variability. These follow-up studies will be essential to substantiate the mechanistic significance and translational potential of Vitamin D3 in modulating Wnt/β-catenin–BDNF signaling under chronic stress.

## Figures and Tables

**Table 1:** Weekly Schedule of Mild Stressors in the 28-Day CMS Protocol (a partially randomized stress model).

Week	Sun	Mon	Tue	Wed	Thu	Fri
1	CT	CS	WB	WD	CL	CL
2	FD	WB	CS	CT	CL	CL
3	CS	WB	WB	WD	CL	CL
4	WB	CT	CT	CS	CL	CL

CMS, Chronic Mild Stress. Stressor abbreviations: CT = Cage Tilting (45° for 24 h), CS = Cage Shaking (150 rpm for 1 h), WB = Wet Bedding (24 h), WD = Water Deprivation (24 h), FD = Food Deprivation (24 h), CL = Continuous Light Exposure (24 h). Stressors were applied in a randomized sequence to induce depressive-like behaviors in rats.

**Table 2:** Primer sequences used for gene expression studies by qPCR.

Gene	Primer Sequence (5′–3′)
Wnt1	F-AACAGTAGTGGCCGATGGTG
	R-GGGTTCTGTCGGATCAGTCG
β-catenin	F-GGCTAACATTCGCCAGTGGA
	R-TGCCACGTCAGCTGGTATAG
GSK-3β	F-ACTCTACCTGAACAGCCCCA
	R-AACGTGACCAGTGTTGCTGA
Axin-2	F-CTCCTTGGAGGCAAGAGC
	R-GGCCACGCAGCACCGCTG
BDNF	F-AGGGAAATCTCCTGAGCCGA
	R-TAATCCAATTTGCACGCCGC

**Table 3:** Changes in body weights at baseline and after the 4-week experimental period in different groups.

Group	Baseline Weight (g)	Final Weight (g)
Control	270.4 ± 5.31	329.3 ± 9.31
CMS	271.7 ± 7.30	300.2 ± 11.04 **^#^**
CMS + vitamin D3 (1000 IU/kg)	274.3 ± 8.77	312.1 ± 7.97 *
CMS + vitamin D3 (10,000 IU/kg)	270.9 ± 5.64	297.0 ± 6.19

Values are presented as mean ± SE (n = 8 per group). Statistical analysis was performed on final weights between groups using one-way ANOVA followed by Holm–Sidak test. *p* < 0.05 was considered statistically significant. Baseline weights showed no significant differences. # indicates *p* < 0.05 in CMS versus control and * indicates *p* < 0.05 for the vitamin D3 (1000 IU/kg) group versus CMS.

## Data Availability

The data is provided within the manuscript.

## Supplementary Materials

The following supporting information can be downloaded at: https://www.mdpi.com/article/10.3390/biomedicines14050977/s1. Table S1: Serum Vitamin D3 and corticosterone levels in control and CMS-exposed rats with Vitamin D3 supplementation.

## Abbreviations

The following abbreviations are used in this manuscript:

CMS	Chronic Mild Stress
Wnt	Wnt (Wingless/Integrated)
GSK-3β	Axin2/glycogen synthase kinase-3β
8-OHdG	8-hydroxy-2′-deoxyguanosine
BNDF	Brain-derived neurotrophic factor
MDA	Malondialdehyde (GSH)
GSH	Glutathione
SOD	Superoxide dismutase
CAT	Catalase
