# Supplementary material for: Interplay of Vitamin D3, Wnt/β-Catenin Pathway, and Oxidative DNA Injury in CMS-Induced Depression Model
Source: Biomedicines. 2026 Apr 24;14(5):977. doi: 10.3390/biomedicines14050977 (PMC13205061; doi:10.3390/biomedicines14050977)
Supplement: Supplementary file 1 [file biomedicines-14-00977-s001.zip › biomedicines-4217563-supplementary.pdf]

Table S1. Serum Vitamin D3 and corticosterone levels in control and CUMS-exposed rats with Vitamin D3 supplementation.

Parameter	Control (Mean ± SD)	CUMS (Mean ± SD)	CUMS + Vit D3 (1000 IU/kg) (Mean ± SD)	CUMS + Vit D3 (10,000 IU/kg) (Mean ± SD)
**Vitamin D3 (ng/ml)**	35.33 ± 2.50	32.52 ± 3.10#	35.17 ± 4.80**	43.86 ± 1.93**
**Corticosterone (ng/ml)**	283.86 ±23.88	426.31 ± 13.06#	390.08 ± 10.93**	276.30± 17.55**

Values are presented as mean ± SD (n = 8 per group). Statistical analysis was performed using one-way ANOVA followed by Holm–Sidak post hoc tests. # indicates $p<0.001$ (CUMS versus control group, and **indicates $p<0.001$ CUMS versus Vitamin D3 treated groups.
